# Natural genetic variability reduces recalcitrance in poplar

**DOI:** 10.1186/s13068-016-0521-2

**Published:** 2016-05-20

**Authors:** Samarthya Bhagia, Wellington Muchero, Rajeev Kumar, Gerald A. Tuskan, Charles E. Wyman

**Affiliations:** Department of Chemical and Environmental Engineering, Bourns College of Engineering, University of California Riverside, 900 University Ave, Riverside, CA 92521 USA; Center for Environmental Research and Technology, Bourns College of Engineering, University of California Riverside, 1084 Columbia Ave, Riverside, CA 92507 USA; Biosciences Division, Oak Ridge National Laboratory, Oak Ridge, TN 37831 USA; BioEnergy Science Center (BESC), Oak Ridge National Laboratory, PO Box 2008 MS6341, Oak Ridge, TN 37831 USA

**Keywords:** Rare poplar variants, Liquid hot water pretreatment, High-throughput pretreatment and co-hydrolysis, Poplar ranks, Lignin comparators

## Abstract

**Background:**

Lignin content and structure are known to affect recalcitrance of lignocellulosic biomass to chemical/biochemical conversion. Previously, we identified rare *Populus trichocarpa* natural variants with significantly reduced lignin content. Because reduced lignin content may lower recalcitrance, 18 rare variants along with 4 comparators, and BESC standard *Populus* was analyzed for composition of structural carbohydrates and lignin. Sugar yields from these plants were measured at 5 process conditions: one for just enzymatic hydrolysis without pretreatment and four via our combined high-throughput hot water pretreatment and co-hydrolysis (HTPH) technique.

**Results:**

Mean of glucan + xylan yields and the best glucan + xylan yield from rare natural poplar variants were 34 and 50 relative percent higher than the high lignin comparator (BESC-316) at the highest severity HTPH condition, respectively. The ability of HTPH to solubilize a large portion of xylan from solids led to small differences in xylan yields among poplar variants. However, HTPH showed large differences in glucan yields, and hence glucan + xylan yields, among the poplar variants. The high lignin comparator did not display lowest glucan + xylan yields with HTPH at moderate pretreatment severity compared to rare variants, but on the other hand, the low lignin comparator was a consistent top performer at all 5 process conditions. Furthermore, the low lignin comparator (GW-11012) showed a 15 absolute percent increase in glucan + xylan yield compared to the high lignin comparator at the most severe HTPH condition. Overall, relative variant rankings varied greatly with pretreatment severity, but poplar deconstruction was significantly enhanced when the pretreatment temperature was increased from 140 and 160 to 180 °C at the same pretreatment severity factor.

**Conclusions:**

Glucan yields from high severity HTPH of rare natural poplar variants with reduced lignin content were significantly higher than from the high lignin comparator. Because of the significant effect of processing conditions on the performance rankings, selection of the best performing biofuel feedstocks should be based on sugar yields tested at conditions that represent industrial practice. From a feedstock perspective, the most consistent variants, SKWE-24-2 and GW-11012, provide key insights into the genetic improvement of versatile lignocellulosic biofuels feedstock varieties.

**Electronic supplementary material:**

The online version of this article (doi:10.1186/s13068-016-0521-2) contains supplementary material, which is available to authorized users.

## Background

Lignocellulosic biomass provides an enormous low-cost source of trapped sugars and aromatics that is the only known option for large-scale production of liquid transportation fuels whose commerce values are so high. However, until reduced greenhouse gas emissions and other externalities are properly recognized and rewarded, commercial success of sustainable fuels and chemicals depends on cost competitiveness with conventional fossil-based oil and gas products. In the case of utilizing lignocellulosic biomass, the challenge to competitiveness is to reduce costs of overcoming its recalcitrance to deconstruction into fermentable sugars [1]. Because plant deconstruction to sugars is essentially the major economic obstacle to producing ethanol from lignocellulosic biomass, less recalcitrant plants would have an economic advantage compared to more recalcitrant counterparts [2]. Accordingly, in recent years, the scientific community has intensified the research into understanding and modifying factors that control recalcitrance of lignocellulosic biomass feedstocks such as poplar and switchgrass. A frequently applied approach is to lower recalcitrance by genetic modification of wild plants to generate superior varieties. An alternative is to identify naturally occurring low recalcitrance varieties by sampling across wide species ranges. Recent advances in linkage disequilibrium-based association genetics and quantitative trait loci (QTL) mapping have made it possible to identify genetic markers for phenotypic traits that affect biomass recalcitrance [3]. These combined approaches have recently shown that some poplar varieties have naturally occurring mutations that lead to reduced lignin biosynthesis [4]. In addition, reducing lignin content through downregulation of lignin biosynthesis enzymes in alfalfa has been previously shown to lower recalcitrance, thus improving sugar yields [2].

Among the many options for valorizing lignocellulosic biomass, enzymatic hydrolysis has been a leading choice for research as well as emerging lignocellulosic biomass commercial-scale processing because of its ability to realize high sugar yields from crystalline cellulose [5, 6]. However, this route typically requires biomass pretreatment prior to enzymatic hydrolysis to recover sugars at high yields essential for low-cost biofuel production. Because coupling these two operations by conventional laboratory methods is time and labor intensive, our previously developed high-throughput pretreatment and co-hydrolysis (HTPH) technique provide a useful tool for rapidly screening large numbers of biomass candidates for sugar yield performance [7]. HTPH pretreats biomass first in a custom-made metal 96-well plate with water at high temperatures, after which, carbohydrate-active enzymes are added to the whole pretreatment slurry to hydrolyze polysaccharides left in the pretreated solids to sugars. In addition to allowing rapid screening of large numbers of biomass materials, the small amounts of biomass required (as low as 10 mg biomass for approximately 3 mg well loadings with 3 replicates) makes it possible to study recalcitrance in the limited quantities often available for experimental plants. Because carbohydrate-active enzymes (CAZys) are strongly inhibited by sugars in the pretreatment liquor as well as those released during enzymatic hydrolysis [8–11], HTPH is generally carried out at a very high loading of CAZys (0.1 g per gram of sugars in biomass) to be sure differences in the digestibility of substrate by enzymes are not masked by enzyme inhibition.

In their study of poplar variants, Studer et al. [12] showed that lignin content as well as syringyl to guaiacyl (S/G) ratio affected sugar yields from natural poplar variants. To build upon these findings, we measured the sugar yield performance of rare poplar varieties with natural genetic alterations in lignin biosynthesis. Eighteen rare poplar variants, four comparators, and the BESC (BioEnergy Science Center) standard poplar were selected for this study. Except for the BESC standard, all were from 4-year-old trees grown and harvested at uniform conditions from Clatskanie, OR as described by Muchero et al. [3]. The BESC standard harvested as a mature tree at Oak Ridge National Laboratory in Tennessee was used as an internal standard to enable comparisons with previously published datasets [7]. The HTPH conditions employed by Studer et al. [12] were adapted as a starting point for our work but extended by adding a higher pretreatment severity condition at a similar CAZy loading. Because lower pretreatment temperatures are desirable to reduce associated costs such as reactor wall thickness and difficulty for biomass feeding at high pressures [13, 14], temperatures of 140, 160, and 180 °C were included over the same severity range to determine if the rare variants could maintain high yields at more economically desirable lower temperatures. With this experimental design, we sought to determine (1) how changes in inherent properties of rare variants, comparators, and standard poplar impact sugar yield trends and (2) the effect of processing conditions on sugar release from a variety of poplar variants with different lignin characteristics. To our knowledge, the resulting study is the first of its kind to report sugar yield performance for a large number of native poplar variants with naturally reduced recalcitrance and determine how rankings of a single species varies with processing conditions of importance in selecting top feedstock candidates for production of biofuels and bioproducts.

## Methods

### Plant materials

Four-year-old *P. trichocarpa* variants were harvested from a field site in Clatskanie, Oregon. Stem segments were immediately dried to constant weight and knife milled through a 20-mesh screen (Model No. 3383-L20, Thomas Scientific, Swedesboro, NJ, USA). Whole stem sections were ground and homogenized thoroughly to avoid variation across different parts of the stems. Details of field establishment and growth conditions are described elsewhere [3, 15]. An additional file provides a list of the poplar variants used in this study (see Additional file 1). The moisture content after milling was an average of 6.75 ± 0.69 % for the variants as determined by a halogen moisture analyzer (HB43-S; Mettler-Toledo, Columbus, OH). The moisture content was taken into account to calculate sugar yields from biomass on a dry basis. The 18 low lignin variants and their 4 comparators were grown in the same field site and were harvested and processed under the same conditions [3]. Comparators were selected to represent the 17.7 to 28.1 % population range of lignin content as reported by Muchero et al. [3]. The BESC standard poplar (BESC STD), a conventional poplar feedstock [7] grown and harvested separately at Oak Ridge National Laboratory, was used as a reference point for comparison with previous publications.

### Compositional analysis

The compositions of unpretreated poplar variants were determined by following the standard NREL LAP “Determination of Structural Carbohydrates and Lignin in Lignocellulosic Biomass [16].” Three technical replicates from the same milled sample were run for each variant.

### Processing conditions

Due to numerous mention of conditions in our results and discussion, from here on we refer to process conditions by their representative alphabetical symbols, similar to those mentioned in Table 1: **A** represents ‘no pretreatment and enzymatic hydrolysis for 120 h’, **B** represents ‘HTPH at 140 °C for 264.4 min (log_10_R_0_ = 3.6)’, **C** represents ‘HTPH at 160 °C for 68.1 min (log_10_R_0_ = 3.6)’, **D** represents ‘HTPH at 180 °C for 17.6 min (log_10_R_0_ = 3.6),’ and **E** represents ‘HTPH at 180 °C for 44.1 min (log_10_R_0_ = 4.0).’ R_0_, the pretreatment severity factor, is defined as t*exp[(T-100)/14.75)], in which t is the pretreatment time in minutes and T is the temperature in  °C [17].

**Table 1:** Hydrothermal pretreatment conditions applied to poplar variants

Condition representative symbol	Condition name	Pretreatment severity factor (log_10_R_0_)^a^	Temperature (°C)	Time (minutes)	Co-hydrolysis enzyme loading	Hydrolysis time (hours)
A	No pretreatment, only enzymatic hydrolysis	N/A	N/A	N/A	75 mg cellulase protein + 25 mg xylanase protein per gram glucan + xylan in raw biomass. Assuming 66.6 % glucan + xylan in biomass	120
B	HTPH 140 °C logR_0_ = 3.6	3.6	140	264.4		24
C	HTPH 160 °C logR_0_ = 3.6	3.6	160	68.1		24
D	HTPH 180 °C logR_0_ = 3.6	3.6	180	17.6		24
E	HTPH 180 °C logR_0_ = 4.0	4.0	180	44.1		24

^a^ R_0_ = t*exp[(T-100)/14.75)], where t is the time in minutes and T is the temperature in  °C. N/A not applicable. log_10_R_0_ is called the pretreatment severity factor [17]

The high-throughput pretreatment and co-hydrolysis (HTPH) procedure was performed as described by Studer et al. [7]. Approximately 4.5 ± 0.15 mg of biomass was loaded into a Hastelloy 96-well plate using either a Freeslate^®^ Core Module II^®^ high-throughput robot equipped with a Sartorius^®^ WZA65-CW balance using powder dispensing methods (Freeslate, Sunnyvale, CA) or with a Mettler-Toledo^®^ MX-5 microbalance (Mettler-Toledo, LLC, Columbus, OH). Each variant had four technical replicates, i.e., four wells were filled with the same sample type (four repeats from the same tree). Four similar plates were prepared for each of the conditions **B** to **E**.

Biomass solid loading was adjusted to be 1 % by adding 445.5 µL of EMD^®^ Millipore^®^ Milli-Q^®^ water. The plates were then sealed, and biomass was allowed to soak overnight at room temperature. The 96-well chassis plates were inserted into our custom-made high pressure chamber that was heated by saturated steam generated in a 75 kW boiler (FB-075-L Fulton Companies, Pulaski, NY) at the appropriate pressure [7]. The steam chamber was pre-heated to the desired temperature for 30 min prior to adding the plates to reduce the pretreatment ramp-up time to 2 to 3 s. When the desired pretreatment time was reached, the steam inlet valve was closed, steam was released from the chamber, and water was quickly filled in the chamber to stop the reaction. The plates were then unsealed, and 30 µL of enzyme mixture was added that contained sodium citrate buffer and sodium azide broad-spectrum antibiotic (Sigma-Aldrich, St. Louis, MO, USA). The sodium citrate buffer and sodium azide concentration in the final solution were 0.05 M and 0.02 %, respectively. The final enzymatic hydrolysis conditions in the well plate were the same as NREL LAP “Enzymatic Saccharification of Lignocellulosic Biomass [18].” An enzyme loading of 75 mg protein of cellulase + 25 mg protein of xylanase per gram glucan + xylan in the unpretreated biomass was applied, assuming all samples had an average of 66.6 % glucan + xylan content (dry basis) in unpretreated poplar. The cellulase used was Accellerase^®^ 1500 (BCA protein content 82 mg/ml), and the xylanase was Accellerase^®^ XY (BCA protein content 51 mg/ml), both generously provided by DuPont Industrial Biosciences, Palo Alto, CA, USA. The protein contents of these commercial preparations were determined by the standard BCA method [19]. Co-hydrolysis was carried out at 50 °C and 150 rpm for 24 h in an incubator shaker (Multitron Infors^®^HT Biotech, Laurel, MD). Enzymatic hydrolysis for condition A (no pretreatment) was carried out in 25 ml Erlenmeyer flasks with a reaction volume of 10 ml, and three technical replicates were run at same conditions for HTPH but for 120 h. The liquors after HTPH or enzymatic hydrolysis for condition **A** were analyzed for sugar concentrations using a Waters^®^ Alliance HPLC (model e2695, Waters Co., Milford, MA) equipped with a Waters^®^ 2414 RI detector and an Aminex^®^ HPX-87H column (Bio-Rad Life Science, Hercules, CA) conditioned at 65 °C with a 5 mM sulfuric acid mobile phase at flow rate of 0.6 ml/min.

### Yield calculations

Sugar yields (glucan, xylan, or glucan + xylan) mentioned throughout this article represent the amount of sugars recovered compared to the amount in unpretreated (raw) biomass (dry basis) on a mass basis, unless otherwise specified. All sugar yields were calculated based on their polymeric form throughout this article using anhydrous factors to account for the mass of water added when polysaccharides are converted into monosaccharides. Thus, the ‘an’ in the sugar names (glucan or xylan) refers to their anhydrous forms. This approach facilitates adding yields directly and closing mass balances as structural carbohydrates (cellulose and hemicellulose) are in their polymeric form in lignocellulosic biomass. The anhydrous factor from glucose (MW of 180.16 g/mol) to glucan is 0.9, and from xylose (MW of 150.13 g/mol) to xylan is 0.88.

The formulae employed are as follows;

$$ {\text{Sugar }}\left( {\text{glycan}} \right)   {\text{yield \%  }}\left( {\text{mg per 100 mg biomass}} \right) = \frac{{\begin{array}{*{20}c} {{\text{Concentration of sugar from HPLC }}\,\,\left( {\frac{\text{mg}}{\text{mL}}} \right) } \\ {* \,{\text{Total volume including moisture in biomass}} \,\left( {\text{mL}} \right) } \\ {* \,0.9 \,{\text{or}} \,0.88 \,{\text{anhydrous factor for glucose or xylose respectively}}} \\ \end{array}  }}{\text{Biomass on a dry basis (mg)}} * 1 0 0 $$

$$ {\text{Absolute change or absolute percent}} = {\text{Sugar Yield \%  of B-Sugar Yield \%  of A}} $$

$$ {\text{Relative percent change}} = \frac{{   {\text{Sugar Yield \%  of B-Sugar Yield \%  of A}}}}{{{\text{Sugar Yield \%  of A}}}} * 1 0 0 $$

### Statistical analysis

Statistical analysis was applied to all comparators and rare variant but not the BESC standard poplar. Thus, the standard poplar data and curve should be viewed as an additional overlay on the data plots. Box plots show the effect of pretreatment on the entire poplar population. The points plotted on the left of each box plot show differences between comparators, rare variants, and standard poplar. An additional file provides more depth in how to interpret the box plots for sugar yields (see Additional file 1). Tukey’s original method of box plots [20], with whiskers extending to maximum and minimum values up to 1.5 times of interquartile range, was used (see Additional file 1). Plots of results were designed on Originlab^®^ v. 6.4 or 2015 statistics and graphing software. Additional file 1 shows the average imprecisions, i.e., the arithmetic mean of standard errors for individual plants.

## Results and discussion

**Fig. 1:**
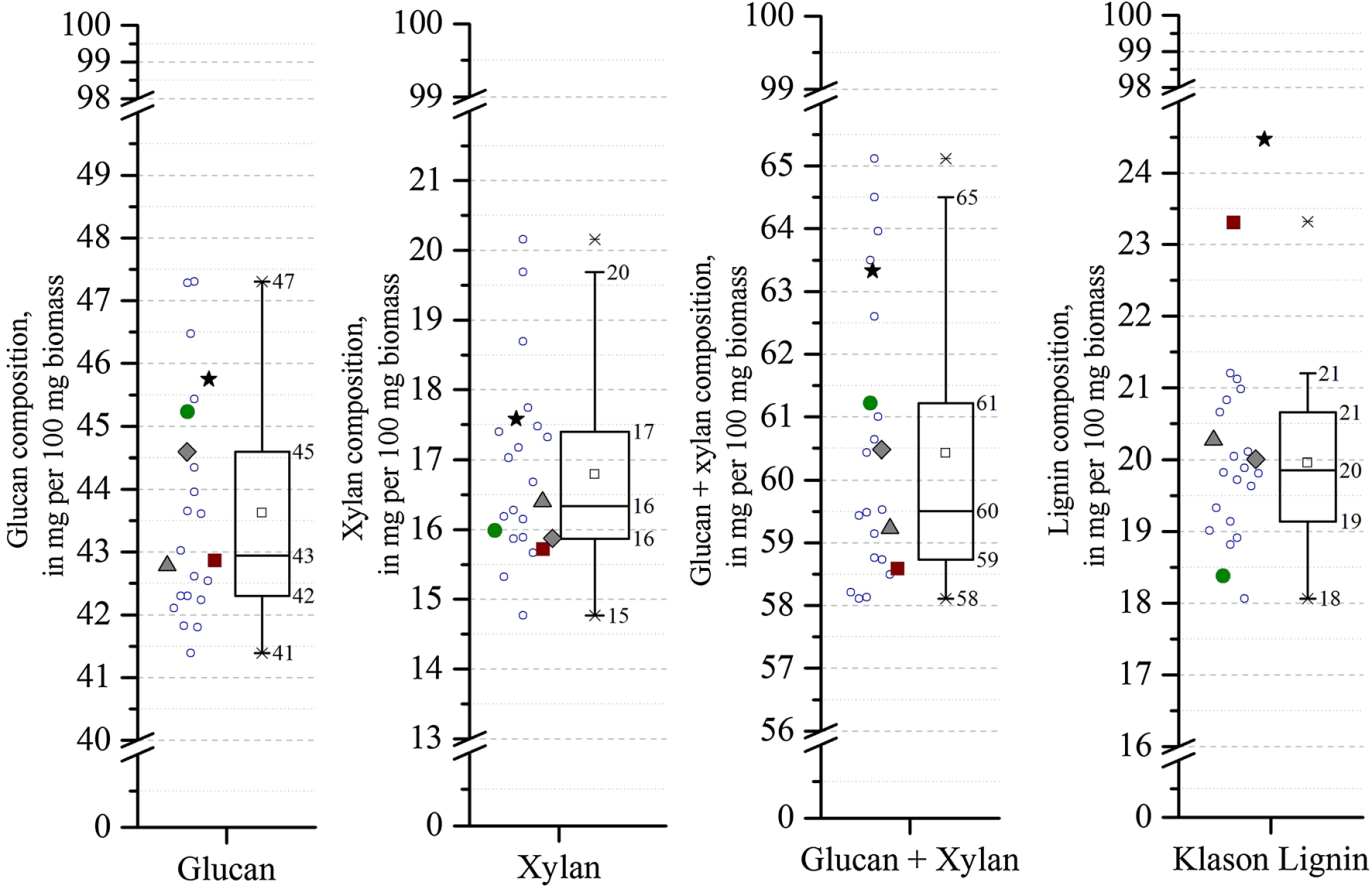
Composition of poplar variants. From *left* to *right*, these *graphs* show glucan, xylan, glucan + xylan, and Klason lignin compositions, respectively, of the raw poplar variants. *Open circles* represent rare variants, *solid circles* represent the low lignin comparator (GW-11012), *solid squares* represent the high lignin comparator (BESC-316), and the *triangle* and *polygon* represent BESC-97 and GW-9762 comparators, respectively. BESC standard poplar is shown with a *solid star*. The *square* inside the box plot represents the mean values of the population. All samples except BESC standard were part of the descriptive box plot statistics

Figure 1 shows the distribution of the mass of structural components glucan, xylan, glucan + xylan, and Klason lignin (acid insoluble lignin) for each of the poplar variants harvested from Clatskanie before processing. The glucan composition ranged from 41 to 47 % with median and mean values of 43 and 44 %, respectively. The xylan contents in unpretreated variants were between 15 and 20 %, with the median and mean being 16 and 17 %, respectively. Taken together, the total glucan + xylan composition ranged from 58 to 65 %, with median and mean values of 59.5 and 60 %, respectively. It is interesting to note that the glucan and xylan amounts varied independently, that is to say, plants with the highest glucan content did not have the highest amount of xylan (data not shown). Also, although *S. cerevisiae* and other yeast can realize high ethanol titers from glucose, native yeast lack the ability to anaerobically metabolize pentose, and even though strains have been developed to co-utilize xylose and glucose [21], a high cellulose content in the raw material can still be beneficial for facilitating conversion to ethanol or other products [22, 23]. The Klason lignin contents of the rare variants ranged between 18 and 21 % and were lower than for the BESC standard poplar (BESC STD). Both the mean and median lignin contents for the rare variants were the same: 19.8 %. On the other hand, the high lignin comparator, BESC-316, had 23.3 % lignin, almost 5 % more than in the low lignin comparator, GW-11012 (18.4 %) in raw biomass. The two other comparators (BESC-97 and GW-9762) had lignin contents toward the lower end of approximately 20 %. BESC STD had the highest lignin contents (24.5 %) among all the plants tested. An additional file presents a bar plot of the differences from the mean of lignin content for all plants (see Additional file 1).

**Fig. 2:**
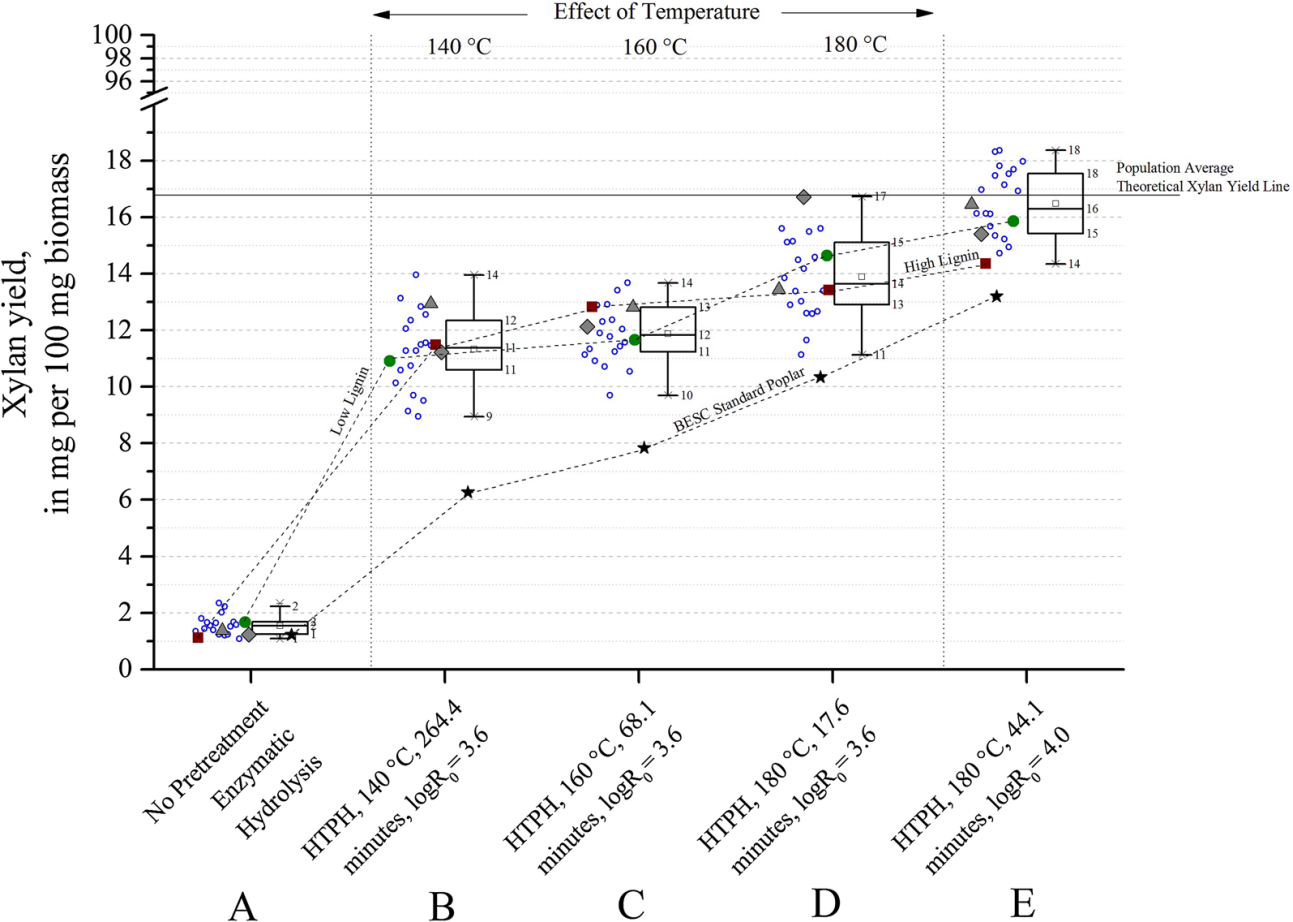
Effect of pretreatment on xylan yields from poplar variants. Five process conditions are shown.* A* sugar release from enzymatic hydrolysis for 120 h without prior pretreatment;* B* HTPH at 140 °C for 264.4 min (logR_0_ = 3.6);* C* HTPH at 160 °C for 68.1 min (logR_0_ = 3.6);* D* HTPH at 180 °C for 17.6 min (logR_0_ = 3.6); and* E* HTPH at 180 °C for 44.1 min (logR_0_ = 4.0). Co-hydrolysis for all HTPH conditions was performed for 24 h at an enzyme loading of 75 mg cellulase protein + 25 mg xylanase protein per gram glucan + xylan in raw biomass. *Open circles* represent rare variants, *solid circles* represent the low lignin comparator (GW-11012), *solid squares* represent the high lignin comparator (BESC-316), and the *triangle* and *polygon* represent BESC-97 and GW-9762 comparators, respectively. BESC standard is shown with a *black star*. The *square* inside the *box plot* represents the mean of the population. All samples except BESC standard were part of the descriptive* box plot* statistics

Figure 2 shows the xylan yields for conditions **A**–**E**. For the entire population, xylan yields from enzymatic hydrolysis without pretreatment (condition **A**) were poor, with the maximum yield of 2 % being achieved with 100 mg total protein loaded per gram glucan + xylan in unpretreated poplar. At any of the HTPH conditions **B** through **E**, there was about 4–6 absolute percent difference between minimum and maximum of xylan yields among samples (not including BESC standard). HTPH conditions **B** (140 °C), **C** (160 °C), and **D** (180 °C) are at the same pretreatment severity factor (logR_0_ = 3.6). Among these conditions, xylan yields increased by 1 absolute percent when the temperature was raised from 140 to 160 °C, and by 2 absolute percent for increasing it from 160 to 180 °C. Near theoretical xylan yields could be achieved when the severity factor was increased from 3.6 to 4.0 (condition **D**–**E**). It is interesting to note that although increasing severity enhanced xylan yields for all samples, the ability of hot water pretreatment to solubilize a large portion of the xylan in all conditions resulted in small differences in xylan yield among samples.

**Fig. 3:**
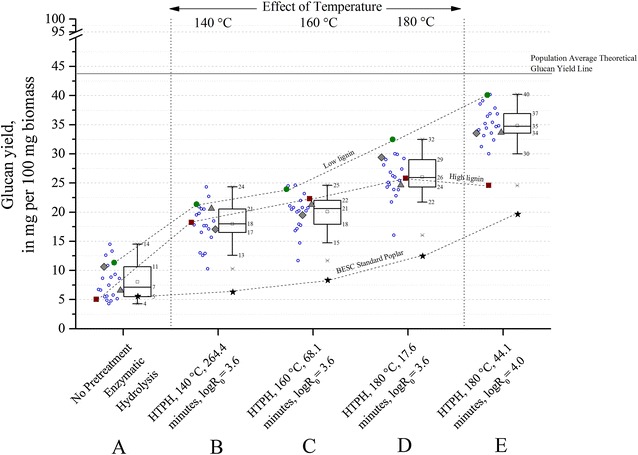
Effect of pretreatment on glucan yields from poplar variants. Five process conditions are shown.* A* sugar release from enzymatic hydrolysis for 120 h without prior pretreatment;* B* HTPH at 140 °C for 264.4 min (logR_0_ = 3.6);* C* HTPH at 160 °C for 68.1 min (logR_0_ = 3.6);* D* HTPH at 180 °C for 17.6 min (logR_0_ = 3.6); and* E* HTPH at 180 °C for 44.1 min (logR_0_ = 4.0). Co-hydrolysis for all HTPH conditions was performed for 24 h at an enzyme loading of 75 mg cellulase protein + 25 mg xylanase protein per gram glucan + xylan in raw biomass. *Open circles* represent rare variants, *solid circles* represent the low lignin comparator (GW-11012), *solid squares* represent the high lignin comparator (BESC-316), and the *triangle* and *polygon* represent BESC-97 and GW-9762 comparators, respectively. BESC standard is shown with a *black star*. The *square* inside the *box plot* represents the mean of the population. All plants except BESC standard were part of the descriptive* box plot* statistics

**Fig. 4:**
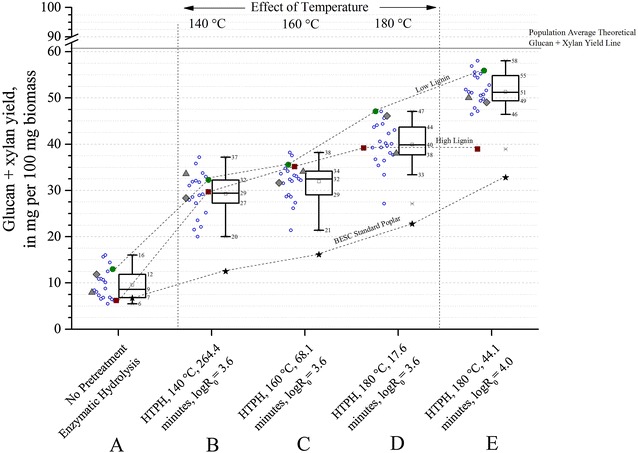
Effect of pretreatment on glucan + xylan yields from poplar variants. Five process conditions are shown.* A* sugar release from enzymatic hydrolysis for 120 h without prior pretreatment;* B* HTPH at 140 °C for 264.4 min (logR_0_ = 3.6);* C* HTPH at 160 °C for 68.1 min (logR_0_ = 3.6);* D* HTPH at 180 °C for 17.6 min (logR_0_ = 3.6); and* E* HTPH at 180 °C for 44.1 min (logR_0_ = 4.0). Co-hydrolysis for all HTPH conditions was performed for 24 h at an enzyme loading of 75 mg cellulase protein + 25 mg xylanase protein per gram glucan + xylan in raw biomass. *Open circles* represent rare variants, *solid circles* represent the low lignin comparator (GW-11012), *solid squares* represent the high lignin comparator (BESC-316), and the *triangle* and *polygon* represent BESC-97 and GW-9762 comparators, respectively. BESC standard is shown with a *black star*. The *square* inside the *box plot* represents the mean of the population. All plants except BESC standard were part of the descriptive box plot statistics

Figures 3 and 4 show that glucan and glucan + xylan yields for 120 h of enzymatic hydrolysis without prior pretreatment (condition **A**) ranged between 4 and 14 % and 6 and 16 %, respectively. The highest 14 % glucan yield and 16 % glucan + xylan yield achieved from the entire population were from one of the rare variants (GW-9920). For comparison of enzymatic hydrolysis yields without pretreatment, the BESC standard poplar had a maximum glucan + xylan yield of 6 %, the high lignin comparator BESC-316 had a similar yield of 6 %, and the low lignin comparator GW-11012 had a yield of 13 %. Thus, these data suggest that some degree of pretreatment is essential for poplar conversion by fungal enzymes to be economically viable, as the best total sugar yield achieved with enzymatic saccharification alone was only 16 % for GW-9920. We can see that solubilization of structural sugars is largely incomplete without pretreatment, even with such a large dose of fungal enzymes. On the other hand, near theoretical yields could be achieved for some of the samples by proper selection of pretreatment conditions (Fig. 4, condition **E**). Thus, the ability of aqueous pretreatments such as hot water and dilute acid to remove most of the xylan, alter lignin, and increase cellulose accessibility substantially improves sugar release by fungal enzymes [24–26].

At any of the conditions **A**–**E,** there was a 10–11 absolute percent and 10–17 absolute percent difference between minimum and maximum of glucan yields (Fig. 3) and glucan + xylan yields (Fig. 4), respectively, among samples (not including BESC standard). These figures show that yields of glucan or glucan + xylan changed little between conditions **B** (HTPH at 140 °C) and **C** (HTPH at 160 °C) that correspond to the same pretreatment severity factor (logR_0_ = 3.6). In particular, the mean and median of glucan yields both increased by 3 absolute percent, and glucan + xylan yields increased by 3 absolute percent when the temperature was raised from 140 to 160 °C at the same severity factor. However, the situation changed when the HTPH temperature was increased to 180 °C (condition **D**) at the same pretreatment severity factor. In this case, raising the temperature from 160 to 180 °C increased the mean and median of glucan yields by 5 absolute percent and glucan + xylan yields by 8 absolute percent. Figure 4 clearly shows that glucan + xylan yields for poplar varieties can easily be increased by 10 absolute percent or more by increasing the temperature to 180 °C (condition **D**) from 140 °C (**B)** or 160 °C (**C**).

These results point to the need to better appreciate the meaning of the pretreatment severity factor that combines time and temperature into a single variable that can facilitate trade-offs between pretreatment time and temperature [17]. A key to this approach is that xylan release from cellulosic biomass can be modeled as a first-order reaction with a rate constant that has an Arrhenius temperature dependence. The value of 14.75 corresponds to the reaction rate doubling with a 10 °C temperature rise [12]. However, the significant increase in sugar yields at 180 °C (condition **D**) compared to 140 °C (condition **B**) and 160 °C (condition **C**), shows that pretreatment severity factor cannot always correctly project how the reaction will respond to varying temperature. This outcome is not surprising in that it would be expected that the bonds between different constituents of the hemicellulose complex would have different activation energies. As a result, the rate constants for breaking these different bonds would not be necessarily expected to all follow the same 10 °C rule, with the outcome that states that yields were higher for pretreatment at 180 °C than at lower temperatures even though the severity factor was the same.

Another important consideration was to understand how yields from the variants responded to an increase in severity factor. In this case, when the severity factor was increased from 3.6 to 4.0 (condition **D** to **E**), overall glucan yields increased by about 10 absolute percent (Fig. 3). As previously mentioned, xylan yields jumped up by virtually 100 % (Fig. 2). As a result, the trends in glucan yield and glucan + xylan yields look somewhat similar at the high severity factor. Because almost all the glucan was conserved in the solids produced by hot water pretreatment at the conditions tested (data not shown) and poplar contains far more glucan than xylan yield, glucan + xylan yields were largely determined by glucan yields from enzymatic hydrolysis. In addition, the fact that xylan yields were virtually 100 % of theoretical for many of the variants at condition **E**, up to 99 % of theoretical glucan + xylan yields could be achieved for one of the rare variants (BESC-35), followed by 94 % from SKWE 24-2.

Figures 2, 3, 4 as well as Tables 2 and 3 show that many of the variants with reduced lignin content can be high sugar-yielding candidates compared to BESC standard poplar. Going in order from conditions **A** to **E,** first with just enzymatic hydrolysis without pretreatment and then with increasing temperature at the same severity and followed lastly by a higher severity at the highest temperature shows that the mean of glucan yields from rare variants increased from 3 to 16 absolute percent compared to standard poplar, the mean of xylan yields for the same variants increased from 1 to 4 absolute percent, and the mean of glucan + xylan yields rose from 3 to 20 absolute percent. It can be seen from Table 3 that the highest glucan + xylan yield from rare variants can be as high as 26 absolute percent and 77 relative percent compared to standard poplar. The relative increase in sugar yields for the rare variants increased in moving to condition **B** (140 °C) followed by conditions **C** (160 °C) and **D** (180 °C) for the same severity and **E** for a higher severity at (180 °C). Both Table 2 and 3 show that within the four HTPH conditions, the relative yield increase dropped in going from condition **B** to **E**. It is important to note that rare variants showed a greater relative increase in sugar yields at lower temperatures than standard poplar.

**Table 2:** Relative and absolute percent changes in the mean of sugar yields among rare variants compared to the high lignin comparator, low lignin comparator, and BESC standard poplar

Condition	Condition details	High lignin comparator			Low lignin comparator			Standard poplar		
		Glucan	Xylan	Glucan + xylan	Glucan	Xylan	Glucan + xylan	Glucan	Xylan	Glucan + xylan
A	No pretreatment enzymatic hydrolysis	59 (3)^a^	44 (1)	56 (4)	−30 (−4)	−6 (−1)	−27 (−4)	44 (3)	30 (1)	42 (3)
B	HTPH 140 °C logR_0_ = 3.6	−3 (−1)	−2 (−1)	−3 (−1)	−18 (−4)	4 (1)	−11 (−4)	181 (12)	80 (5)	131 (17)
C	HTPH 160 °C logR_0_ = 3.6	12 (−3)	−9 (−2)	−11 (−4)	−18 (−5)	1 (1)	−12 (−5)	137 (12)	51 (4)	95 (16)
D	HTPH 180 °C logR_0_ = 3.6	−2 (−1)	3 (1)	1 (1)	−22 (−7)	−7 (−1)	−17 (−8)	106 (14)	33 (4)	73 (17)
E	HTPH 180 °C logR_0_ = 4.0	44 (11)	17 (3)	34 (14)	−13 (−5)	6 (1)	−8 (−4)	80 (16)	27 (4)	59 (20)

^a^ Values in parentheses represent absolute change

**Table 3:** Relative and absolute percent changes in best sugar yields among rare variants compared to the high lignin comparator, low lignin comparator, and BESC standard poplar

Condition	Condition details	High lignin comparator			Low lignin comparator			Standard poplar		
		Glucan	Xylan	Glucan + xylan	Glucan	Xylan	Glucan + xylan	Glucan	Xylan	Glucan + xylan
**A**	No pretreatment enzymatic hydrolysis	188 (10)^a^	111 (2)	161 (10)	29 (4)	39 (1)	24 (4)	161 (9)	92 (2)	137 (10)
**B**	HTPH 140 °C logR_0_ = 3.6	34 (7)	22 (3)	26 (8)	14 (3)	29 (4)	16 (5)	287 (19)	123 (8)	196 (25)
**C**	HTPH 160 °C logR_0_ = 3.6	11 (3)	7 (1)	9 (4)	3 (1)	18 (3)	8 (3)	196 (17)	75 (6)	137 (23)
**D**	HTPH 180 °C logR_0_ = 3.6	17 (5)	17 (3)	17 (7)	−8 (−3)	7 (1)	−4 (−2)	141 (18)	51 (6)	100 (23)
**E**	HTPH 180 °C logR_0_ = 4.0	64 (16)	29 (5)	50 (20)	1 (1)	16 (3)	4 (3)	105 (21)	40 (6)	77 (26)

^a^ Values in parentheses represent absolute change

As shown in Tables 2 and 3, the mean and maximum glucan + xylan yields from rare variants increased by a relative 34 and 50 %, respectively, compared to the high lignin comparator (BESC-316) at condition **E**. For the high lignin comparator BESC-316, low glucan and glucan + xylan yields were only clearly evident at condition **A**, where yields were less than the 25 % percentile, and condition **E** where it was an outlier. For conditions **B**, **C,** and **D** that all were at a logR_0_ of 3.6, its yields were not the lowest in the population. At condition **B**, the yield of the high lignin comparator was close to the population median. At condition **C,** its yield rose above the 75 percentile and dropped down to close to the median (50 % percentile) at condition **D**. Some of the rare variants had significantly lower sugar yields than the high lignin comparator at these conditions. The box plots in Figs. 3 and 4 show that glucan and glucan + xylan yields for the low lignin comparator (GW-11012) were above the 75 percentile of the population for all conditions **A**–**E**. The absolute and relative change in the mean of glucan + xylan yields from rare variants were negative compared to the low lignin comparator (GW-11012) (Table 2), as GW-11012 had one of the highest glucan + xylan yields (Fig. 4). At conditions **D** and **E,** it ranked highest in glucan yield among all samples. However, some of the rare variants gave 4 to 24 % higher relative glucan + xylan yields than the low lignin comparator (Table 3). The same three conditions in Fig. 3 show that the differences in glucan yields between low and high lignin comparator were only 2 to 3 absolute percent at the lower temperatures **B** (140 °C) and **C** (160 °C) but increased to 7 absolute percent at **D** (180 °C). At the higher severity factor condition **E**, this difference increased to 15 absolute percent. Going back to the plot for the distribution of Klason lignin content in Fig. 1, we see that lignin contents for rare variants and three comparators (GW-11012, BESC-97, and GW-9762) are within a range of 3 absolute percent. These samples with only a 3 absolute percent difference in lignin content were found to have a 10 absolute percent variation in glucan + xylan yields. One of the rare variants (CHWH-27-2, Additional file 1) had a lignin content lower than but close to the low lignin comparator (GW-11012). Thus, although these rare variants might share common mutations, variation in sugar yields can be attributable to other genetic differences such that factors other than lignin content alone have significant effects on deconstruction.

Standard poplar had an almost 5 absolute percent higher lignin content (24.5 %) than the mean of lignin contents of the rare variants (19.8 %) but was similar to that for the high lignin comparator BESC-316 (23.3 %). In fact, BESC standard poplar showed such a low sugar yield that statistical analysis found it to be an outlier at all conditions compared to any of the variants, including the comparators. One would expect that if only lignin content affected sugar yields, then BESC-316 and BESC STD would exhibit similar yields, but this was clearly not the case as the glucan + xylan yields for BESC-316 were 6 to 19 absolute percent higher than for BESC STD for the full range of HTPH conditions **B**–**E**.

Since the BESC STD was a much older plant at harvest and grown at a different site than all other poplar variants in this study (rare variants + comparators), these observations suggest that environmental factors, harvest age, growth site, harvest season, climate, and soil conditions could affect phenotypic plasticity and cause significant difference cell wall characteristics and subsequent sugar yields [27, 28]. Although standard poplar is not a true comparator to the variants, the differences in yields still point to an important result: there can be extreme differences in sugar yields within the same species, possibly due to differences in age or phenotypic plasticity. However, the significant differences in performance for samples grown, harvested, and processed under the same conditions suggest that genetic variability confers major differences in cell wall recalcitrance and provides opportunities for improving lignocellulosic biofuel feedstocks.

As noted earlier, xylan yields did not change noticeably with plant variety in that a high percentage of xylan was always recovered. Thus, as shown in Fig. 2, trends in xylan yields from low to high lignin comparators was unclear for lower temperature pretreatments at a severity parameter of 3.6 (conditions **B** and **C**). In fact, the high lignin comparator BESC-316 produced about a 1 absolute percent higher xylan yield than the low lignin comparator GW-11012 for these two conditions. However, for both low severity (condition **D**) and high severity (condition **E**) pretreatments at 180 °C, the low lignin comparator had about 1 absolute percent higher xylan yields than from the high lignin comparator. Xylan yields as a percentage of the theoretical maximum were 85 and 91 % from the high and low lignin comparators, respectively, at condition **D** and increased to 91 and 99 %, in that order, at condition **E**. Overall, glucan + xylan yields were much greater from the low lignin comparator than from the high lignin comparator due to larger differences in glucan yields rather than xylan yields. At the most severe HTPH (condition **E**), the theoretical glucan + xylan yields ranged from 73 to 99 % for the rare variants (data not shown), but the range dropped to 47 to 76 % (data not shown) at the lower severity factor condition **D** even though the temperature was the same. The two comparators, GW-9762 and BESC-97, had lignin contents and sugar yields similar to the rare variants.

**Fig. 5:**
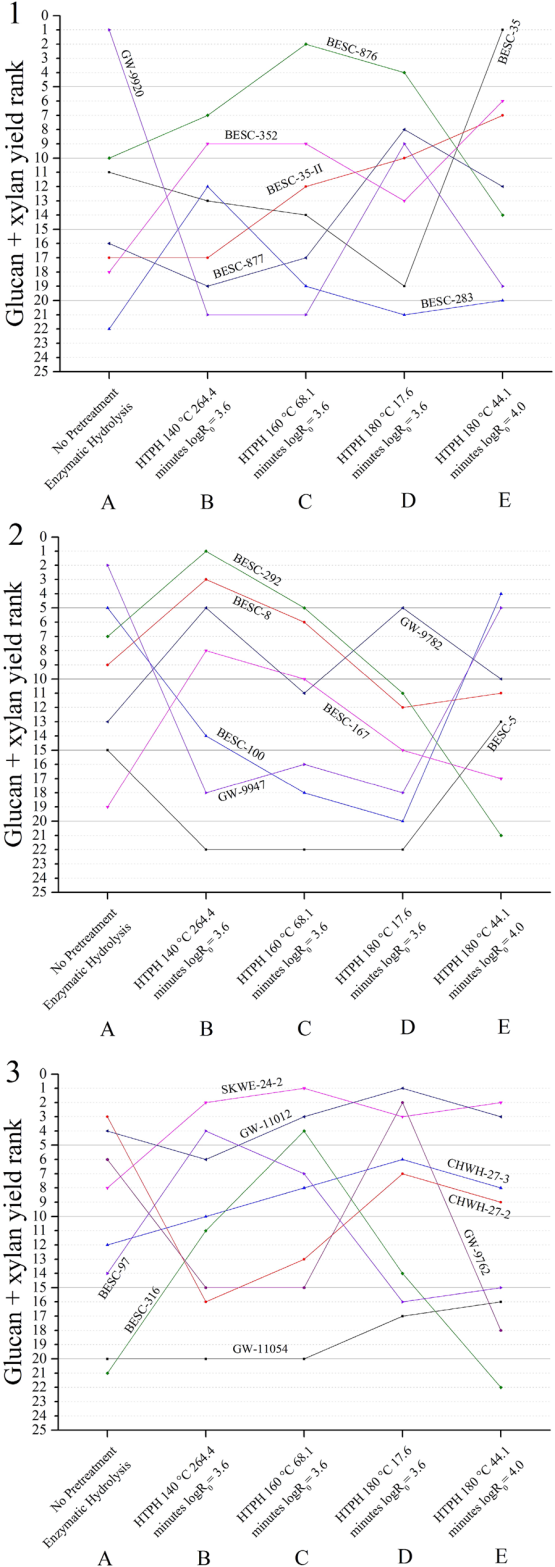
*Graphs* 1, 2, and 3 represent the total glucan + xylan yield ranks for 22 variants from at different processing conditions. The highest yield is rank as 1, and the lowest yield is ranked as 22. Conditions from *left* to *right* are (*1*) enzymatic hydrolysis for 120 h without prior pretreatment, (*2*) HTPH at 140 °C for 264.4 min (logR_0_ = 3.6), (*3*) HTPH at 160 °C for 68.1 min (logR_0_ = 3.6), (4) HTPH at 180 °C for 17.6 min (logR_0_ = 3.6), and (5) HTPH at 180 °C for 44.1 min (logR_0_ = 4.0). Enzymatic hydrolysis for all HTPH experiments was applied for 24 h. The enzyme loading for all conditions was 75 mg cellulase protein + 25 mg xylanase protein per gram glucan + xylan in the raw biomass

Figure 5 summarizes the overall ranking of glucan + xylan yields for all the samples included in this study. We can clearly see that glucan, xylan, and glucan + xylan yield ranks changed drastically with process conditions. For example, GW-9920 ranked 1st in glucan + xylan yield for enzymatic hydrolysis without prior pretreatment (condition **A**) but dropped to 19th out of 22 total for high severity pretreatment at 180 °C (condition **E**). On the other hand, the glucan + xylan yield rank of BESC-35 rose from 11 to 1 when moving from condition **A** to condition **E**. Thus, we can see that within the range of pretreatment conditions applied, severity factor and temperature both impacted sugar yield ranks. Low lignin comparator GW-11012 was one of the more consistent performers in that glucan + xylan ranks stayed between 1 and 6 out of 22 for all five process conditions. Rare variant SKWE 24-2 was also a consistent top performer as it displayed a high rank among the pretreatment conditions but average without pretreatment.

Figure 5 also shows that although CHWH-27-2 produced 3rd from the best yields when enzymatically hydrolyzed without prior pretreatment (condition **A**), its performance fell to 9th when subjected to the highest severity pretreatment prior to enzymatic hydrolysis (condition **E**). On the other hand, the yield from the low lignin comparator GW-11012 rose from the 4th rank without pretreatment (condition **A**) to 3rd rank at for the most severe pretreatment (condition **E**). This shift in performance strongly suggests that factors other than lignin content have a major impact on sugar yields. It is important to remember that the lignin wall in lignocellulosic biomass can be viewed in terms of three major characteristics: lignin amount, lignin strength, and lignin location. For the first, high lignin contents have been shown to negatively affect sugar yields [12]. Strength, the second characteristic, depends on several underlying factors that affect nature of bonding. For one, the ratio of syringyl to guaiacyl units (S/G) affects the strength of lignin bonds in that the higher S/G ratios increase carbon to oxygen (β-O-4) linkages that are more susceptible to breakdown than carbon to such carbon bonds as β-β, β-5, β-1, and 5-5 [29, 30]. Interactions with carbohydrates in lignin-carbohydrate complexes (LCC) also affect lignin strength [31]. The third category, location, refers to lignin distribution in native plant cell walls. Complex bond cleavage and re-association reactions occur in lignin when biomass is subjected to such high temperature processes as aqueous pretreatment. These reactions alter lignin distribution and are thought to move lignin from the interior of cell walls to the outer surface where it can still affect enzymatic deconstruction of cellulose [32, 33]. The shift in one or more of these lignin attributes might be responsible for the high lignin comparator showing poor relative performance without pretreatment (condition **A**) and for high severity HTPH at 180 °C (condition **E**) while still performing relatively well when pretreated at moderate severity conditions (**B**, **C,** and **D**). This result also suggests that high lignin content may not universally result in very low yields within plant populations.

These findings indicate that the optimum processing conditions can vary considerably even for a single species of plants. That is to say, plants that perform well without pretreatment may not perform best with pretreatment because yields are too low to be economically attractive without pretreatment for all the poplar varieties tested with fungal enzymes. Thus, making choices based on results from enzymatic hydrolysis without pretreatment is likely to eliminate feedstocks that have the greatest yield potential. Therefore, screening of plants for top candidates for cellulosic ethanol or biomass-derived products should be based on tests that mimic processing conditions to be applied in a commercial-scale biofuels plant.

## Conclusions

There was an overall 10–17 absolute percent difference between minimum and maximum glucan + xylan yields for poplar variants at all process conditions. The mean of glucan + xylan yields and the best glucan + xylan yield from rare natural poplar variants were 34 and 50 relative percent higher than the high lignin comparator at the highest severity HTPH condition, respectively. Thus, these plants promise to be superior feedstocks for fungal enzyme processing into sugars that can be fermented into ethanol or other products. However, sugar yields from all poplar variants were higher for liquid hot water pretreatment applied at 180 °C than at 140 or 160 °C, even though the severity was held constant. Although the high recovery of xylan from all samples by HTPH resulted in small differences in xylan yields among poplar variants, there were large differences in glucan yields among poplar variants with HTPH. The negative effect of high lignin content on glucan + xylan yield was clear with HTPH at high severity but not at moderate severity. The low lignin comparator had one of the highest sugar yields for all process conditions. Moreover, the low lignin comparator achieved a 15 absolute percent increase in glucan + xylan yields compared to the high lignin comparator at the more severe 180 °C HTPH condition. Overall, the large variance in poplar variant ranks with processing conditions shown here indicates that the choice of poplar feedstocks should be based on tests that mimic expected operating conditions and not based on performance without pretreatment or fully optimized conditions.

## Additional file

10.1186/s13068-016-0521-2Additional details for rare natural poplar variants. Additional file 1 provides the list of plants and their lignin content. This file also provides details of the graphs with box plots, and average imprecisions in measurement of sugar yield and lignin content.

## Abbreviations

HTPH: high-throughput hot water pretreatment and co-hydrolysis
BESC: Bioenergy Science Center
BESC STD: BESC standard poplar
