# Supplementary material for: Natural genetic variability reduces recalcitrance in poplar
Source: Biotechnol Biofuels. 2016 May 20;9:106. doi: 10.1186/s13068-016-0521-2 (PMC4874023; doi:10.1186/s13068-016-0521-2)
Supplement: Supplementary file 1 — 10.1186/s13068-016-0521-2Additional details for rare natural poplar variants. Additional file 1 provides the list of plants and their lignin content. This file also provides details of the graphs with box plots, and average imprecisions in measurement of sugar yield and lignin content. [file 13068_2016_521_MOESM1_ESM.docx]

**Identification of key levers for reducing recalcitrance in poplar**

Samarthya Bhagia^1,2,4^, Muchero Wellington^3,4^, Rajeev Kumar^2,4^, Gerald A. Tuskan^3,4^, and Charles E. Wyman^1,2,4^

^1^Department of Chemical and Environmental Engineering, Bourns College of Engineering, University of California Riverside, 900 University Ave, Riverside, CA 92521, USA

^2^Center for Environmental Research and Technology, Bourns College of Engineering, University of California Riverside, 1084 Columbia Ave, Riverside, CA 92507, USA

^3^Biosciences Division, Oak Ridge National Laboratory, Oak Ridge, TN 37831, USA

^4^BioEnergy Science Center (BESC), Oak Ridge National Laboratory, PO Box 2008 MS6341, Oak Ridge, TN 37831, USA

**Additional Table 1.** List of poplar variants

| **Field Sites** | **Clatskanie, OR** |
| --- | --- |
| 1 | BESC-35 |
| 2 | BESC 35-II |
| 3 | BESC-283 |
| 4 | BESC-352 |
| 5 | BESC-876 |
| 6 | BESC-877 |
| 7 | GW-9920 |
| 8 | BESC-5 |
| 9 | BESC-8 |
| 10 | BESC-100 |
| 11 | BESC-167 |
| 12 | BESC-292 |
| 13 | GW-9782 |
| 14 | GW-9947 |
| 15 | GW-11054 |
| 16 | CHWH-27-2 |
| 17 | CHWH-27-3 |
| 18 | SKWE-24-2 |
|  |  |
| Comparators | |
| **High Lignin** | BESC-316 |
| **Low Lignin** | GW-11012 |
|  | BESC-97 |
|  | GW-9762 |

**
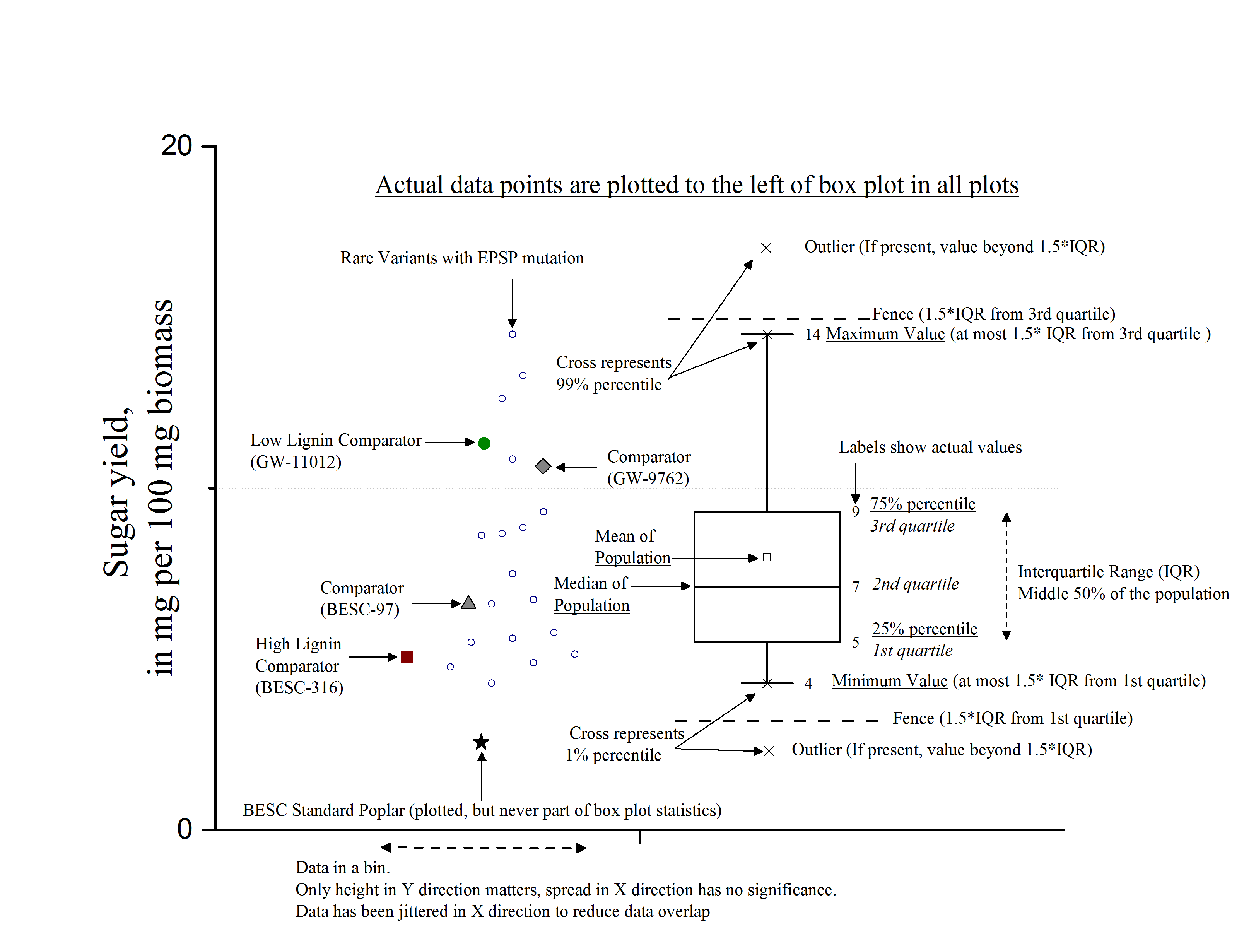
**

**Additional Figure 1.** Understanding box plots used in this study.

**Additional Table 2.** Average imprecision % in measurement.

| Condition |  | Glucan | Xylan | Glucan + Xylan | Lignin |
| --- | --- | --- | --- | --- | --- |
|  | Composition | 0.19 | 0.06 | 0.20 | 0.33 |
| A | No pretreatment enzymatic hydrolysis | 0.13 | 0.05 | 0.14 | N/A |
| B | HTPH 140°C logR_0_ = 3.6 | 0.59 | 0.35 | 0.69 | N/A |
| C | HTPH 160°C logR_0_ = 3.6 | 0.72 | 0.37 | 0.81 | N/A |
| D | HTPH 180°C logR_0_ = 3.6 | 0.97 | 0.42 | 1.05 | N/A |
| E | HTPH 180°C logR_0_ = 4.0 | 0.49 | 0.23 | 0.54 | N/A |

N/A- not applicable


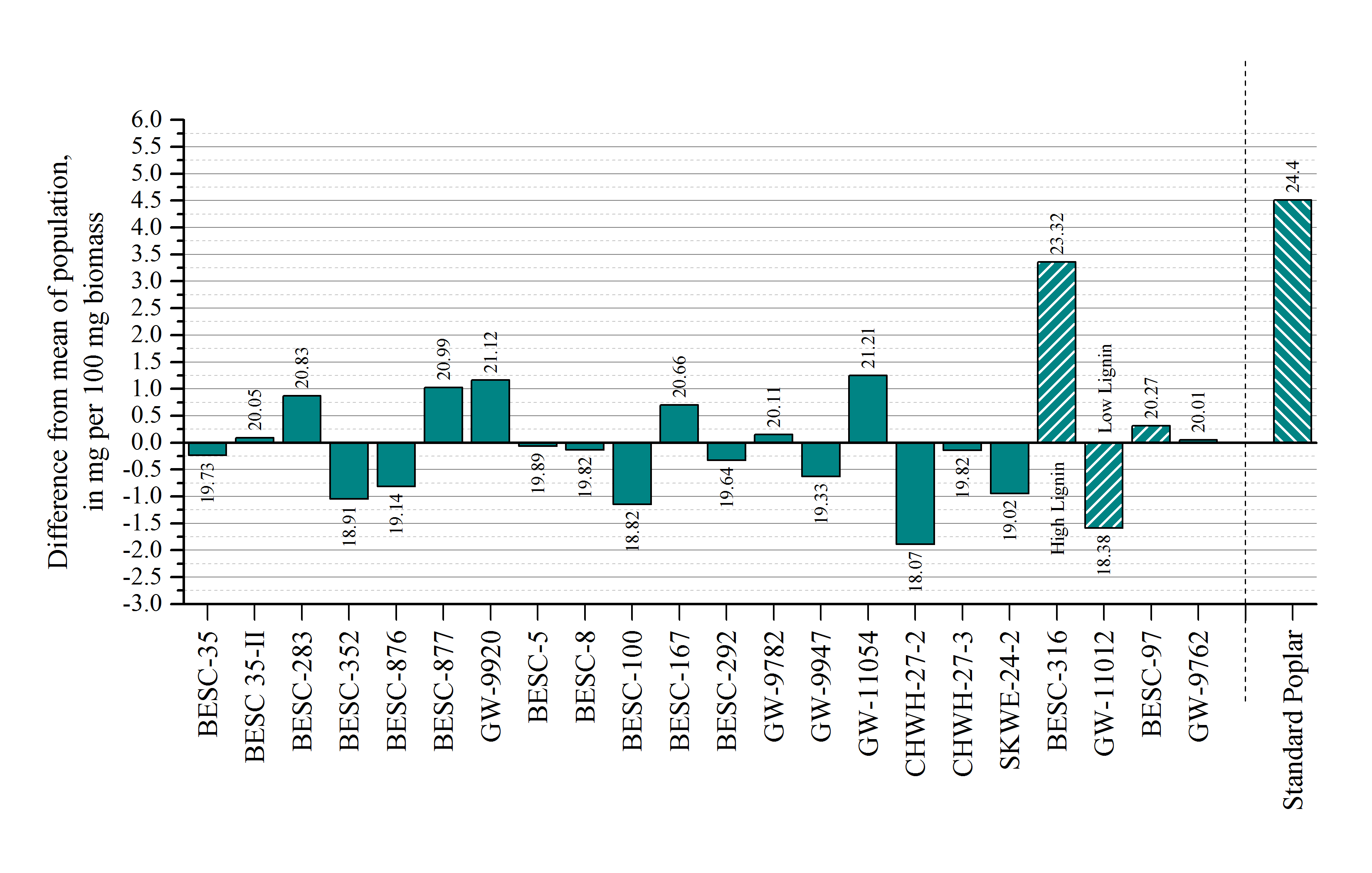


**Additional Figure 2.** Klason lignin composition of Clastkanie plants and BESC standard poplar, units in mg per 100 mg biomass (dry basis). Columns are represented as percent relative difference from mean of the population. Column data labels show actual Klason lignin values. Population mean is 19.96%. Average imprecision in measurement is 0.35%. Columns with pattern are comparators and do not have the natural mutation. BESC standard poplar, shown for comparison, was not part of the statistic, and was not grown in Clatskanie, OR.
